# Ferroptosis-immune-metabolic axis in asthma: mechanistic crosstalk, endotype-specific regulation, and translational targeting

**DOI:** 10.3389/fimmu.2025.1714608

**Published:** 2026-01-07

**Authors:** Feng-Xian Ni, Jie Hu, Pei-Sheng Chen, Hui-Hui Chen, Dong-Hui Huang, Ze-Bo Jiang

**Affiliations:** 1 Zhuhai Hospital of Integrated Traditional Chinese & Western Medicine, Zhuhai, Guangdong, China; 2 Zhuhai Hospital Affiliated to Faculty of Chinese Medicine, Macau University of Science and Technology, Zhuhai, Guangdong, China

**Keywords:** ferroptosis, asthma, ferroptosis-immune-metabolic axis, airway inflammation, endotype-specific therapy, precision medicine

## Abstract

Asthma, a heterogeneous chronic respiratory disorder affecting millions globally, is driven by complex interactions between genetic susceptibility, environmental triggers, and dysregulated immunity. Emerging evidence positions ferroptosis, an iron-dependent form of regulated cell death characterized by lipid peroxidation, as a pivotal mechanism. This review introduces the “Ferroptosis-Immune-Metabolic Axis” as an integrative framework for asthma pathogenesis. We detail how environmental insults (e.g., allergens, pollutants) initiate ferroptosis in airway epithelial cells, leading to the release of damage-associated molecular patterns (DAMPs) and lipid peroxidation products (e.g., 4-HNE). These molecules activate and recruit immune cells (M1 macrophages, neutrophils, Th17 cells), which in turn exacerbate oxidative stress and iron dysregulation, creating a self-amplifying cycle. Metabolic reprogramming, including enhanced polyunsaturated fatty acid (PUFA) synthesis and glycolytic flux, provides the essential substrates and energy to sustain this vicious cycle. We dissect endotype-specific manifestations: IL-33-driven epithelial ferroptosis in eosinophilic asthma and ALOX15-mediated lipid peroxidation coupled with hepcidin-induced iron retention in neutrophilic asthma. Therapeutically, we highlight novel strategies such as inhaled GPX4 mRNA nanocarriers and ALOX15 inhibitors, underscoring the potential of targeting this axis for precision medicine in refractory asthma.

## 1 Introduction

Asthma represents a significant global health burden, characterized by chronic airway inflammation, hyperresponsiveness, and remodeling. While type 2 inflammation is well-characterized, many patients, particularly those with severe or neutrophilic phenotypes, respond poorly to current therapies, necessitating deeper insights into alternative pathogenic mechanisms (1, 2). Ferroptosis, a regulated cell death pathway driven by iron-dependent phospholipid peroxidation, has recently emerged as a critical player in asthma pathogenesis (3). Distinct from apoptosis or necrosis, ferroptosis features unique morphological characteristics, including mitochondrial shrinkage, plasma membrane rupture, and toxic lipid peroxide accumulation (4). Its execution is regulated by metabolic and signaling components, such as glutathione peroxidase 4 (GPX4), the cystine/glutamate antiporter (system Xc^-^), and lipid metabolism enzymes (5). Dysregulation of these pathways can amplify airway inflammation and tissue damage.

The immune system orchestrates inflammatory cascades in asthma, involving macrophages, neutrophils, T cells, and eosinophils. These cells release pro-inflammatory cytokines and reactive oxygen species (ROS), exacerbating oxidative stress and recruiting additional immune cells to the lungs (6). The interplay between ferroptosis and immune dysregulation is bidirectional: oxidative stress accelerates ferroptosis, which in turn promotes the release of damage-associated molecular patterns (DAMPs) from dying cells. These DAMPs activate immune responses, creating a self-sustaining cycle of inflammation and cellular damage (7). This feedback mechanism may contribute to the chronicity and severity of asthma, particularly in neutrophilic subtypes (8).

**Figure 1:**
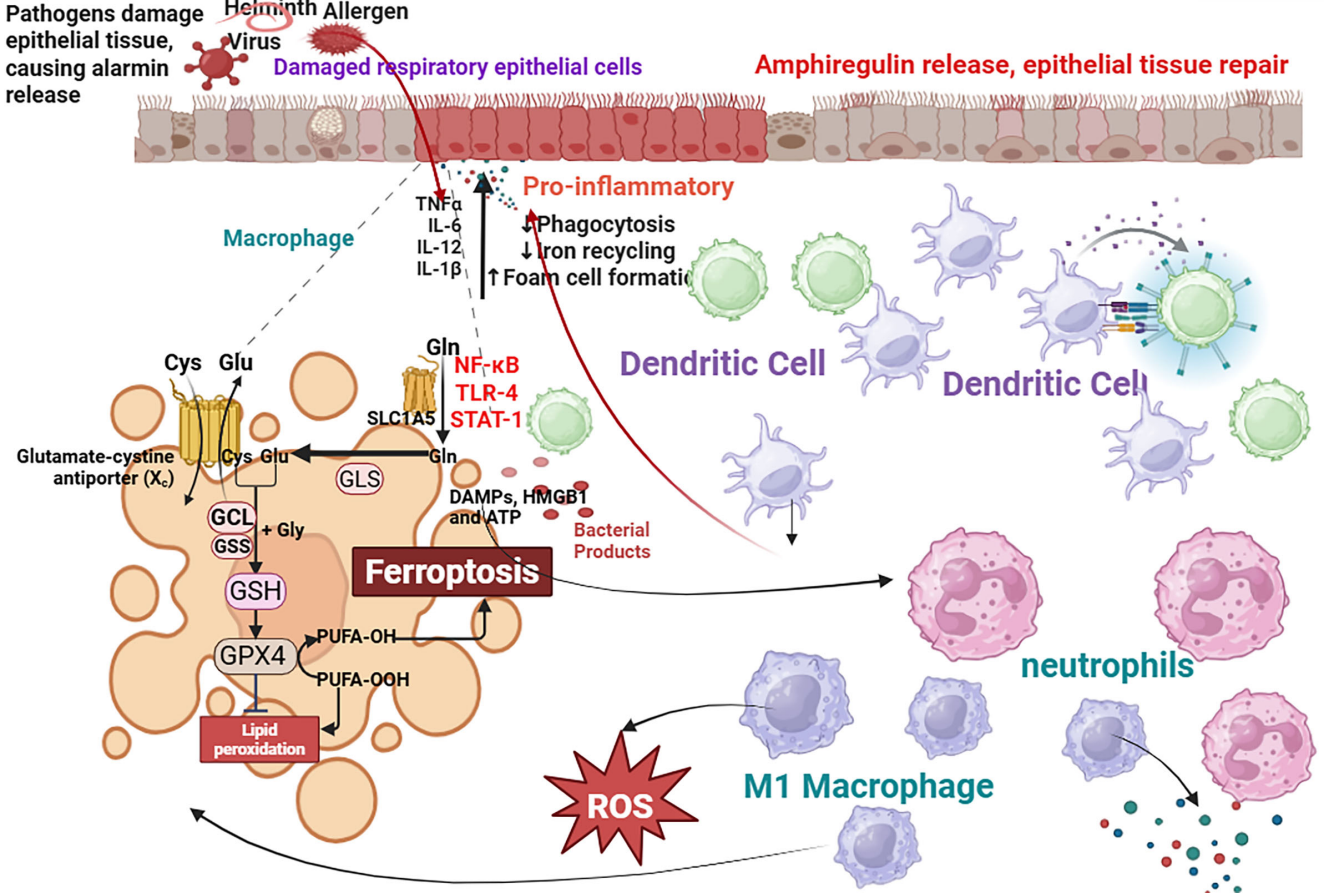
The vicious cycle of ferroptosis and immune dysregulation in asthma. Environmental triggers (allergens, pollutants) initiate ferroptosis in airway epithelial cells through mitochondrial dysfunction, ER stress, and iron overload. Ferroptotic cell death leads to the release of damage-associated molecular patterns (DAMPs) such as HMGB1 and ATP, and lipid peroxidation products like MDA and 4-HNE. These signals activate innate immune cells (e.g., macrophages, neutrophils) and adaptive immune cells (e.g., Th2, Th17). Activated M1 macrophages and neutrophils further exacerbate oxidative stress and lipid peroxidation through ROS production and expression of enzymes like ALOX15, creating a pro-ferroptotic microenvironment. They also secrete pro-inflammatory cytokines (IL-6, TNF-α, IL-17A) that can directly inhibit antioxidant defenses (e.g., GPX4) in structural cells. This bidirectional crosstalk establishes a self-perpetuating cycle of epithelial injury, sustained inflammation, and airway remodeling, characteristic of severe asthma. Anti-inflammatory M2 macrophages and Tregs, which normally resolve inflammation and suppress ferroptosis, are impaired. Created with BioRender.

To systematically describe these interactions, we propose the “Ferroptosis-Immune-Metabolic Axis” in asthma. As illustrated in **Figure 1**, ferroptosis-derived signals such as lipid peroxides (e.g., MDA and 4-HNE) and DAMPs (e.g., HMGB1, IL-33) activate immune cells, including M1 macrophages and Th17 cells. Activated immune cells release cytokines (e.g., IL-6, TNF-α) and ROS, further disrupting iron homeostasis (e.g., via IRP2 activation and ferritinophagy) and promoting lipid remodeling (e.g., through ACSL4 upregulation), thereby amplifying ferroptosis. Underpinning this crosstalk is metabolic reprogramming—such as enhanced glycolysis in macrophages and increased polyunsaturated fatty acid (PUFA) synthesis in epithelial cells—which supplies the substrates necessary for both ferroptosis and immune activation, closing a vicious cycle.

Beyond mediating cell death, ferroptosis influences immune function by modulating cytokine profiles and cell recruitment (9). DAMPs released during ferroptosis enhance inflammatory cells infiltration into the airways, sustaining a pro-inflammatory microenvironment and contributing to tissue remodeling (10). A deeper understanding of this axis is critical for unraveling asthma pathophysiology and revealing novel therapeutic opportunities. Recent studies have explored targeting ferroptosis as a potential treatment strategy, especially in refractory asthma (11). Pharmacological inhibitors of ferroptosis and compounds that enhance cellular antioxidant capacity represent promising avenues. Additionally, identifying ferroptosis-associated biomarkers could facilitate patient stratification and personalized treatment approaches (12). This review provides a comprehensive analysis of the interplay between ferroptosis, immune dysregulation, and metabolic reprogramming in asthma. We detail the underlying molecular mechanisms, discuss their implications for disease progression, and highlight emerging therapeutic strategies aimed at disrupting this maladaptive axis. By integrating recent advances, we aim to clarify the role of ferroptosis in asthma and stimulate further research into its therapeutic potential.

## 2 Core machinery of ferroptosis in the asthma context

Ferroptosis is governed by a balance between pro-ferroptotic (iron overload, lipid peroxidation) and anti-ferroptotic (antioxidant defense) pathways. In asthma, this balance is tilted toward ferroptosis by axis activation-below we focus on key molecules and pathways relevant to airway pathophysiology.

### 2.1 Definition systems: the GPX4 and FSP1 armadas

**Figure 2:**
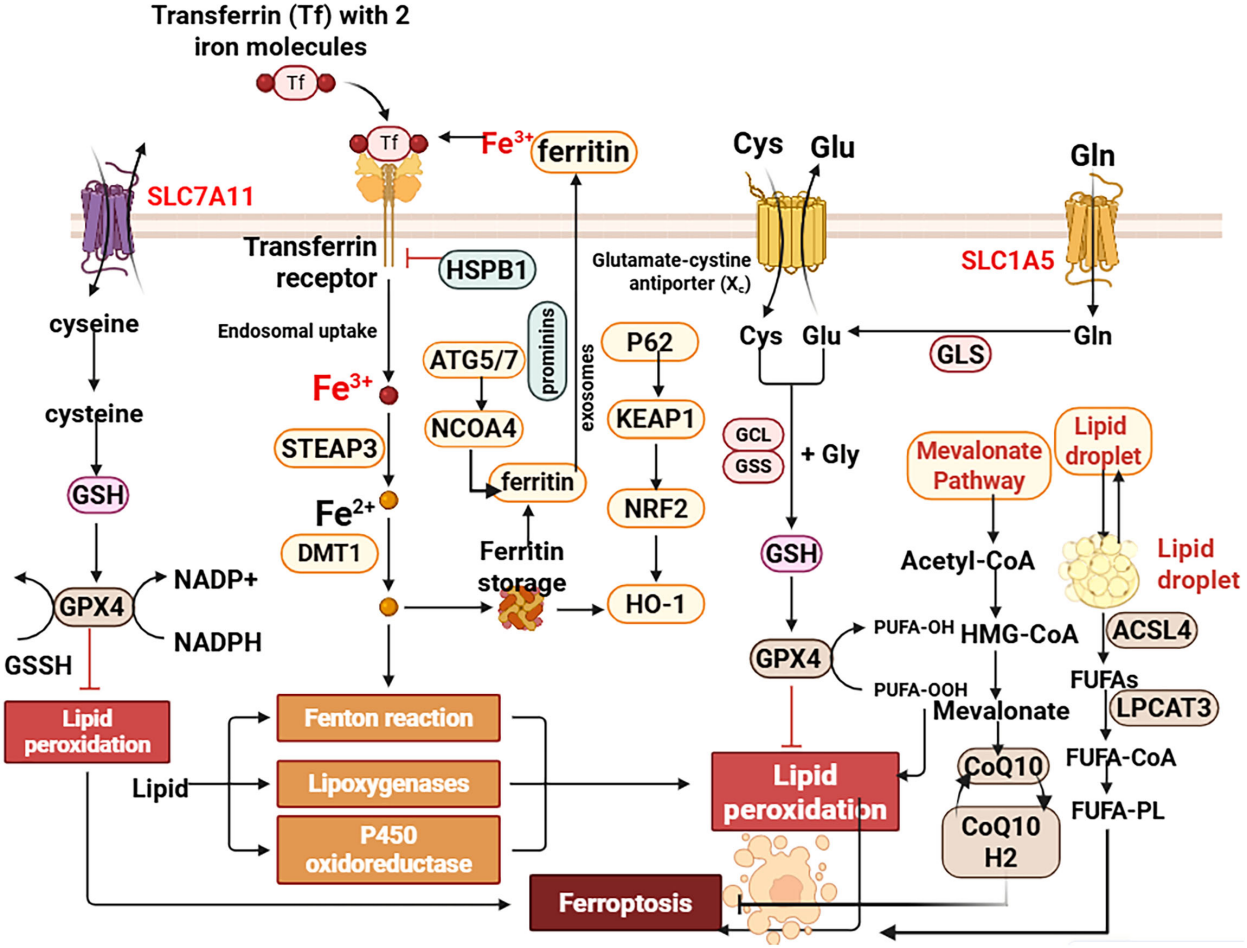
Molecular mechanisms of ferroptosis. Ferroptosis is an iron-dependent form of regulated cell death driven by the peroxidation of polyunsaturated fatty acid (PUFA)-containing phospholipids. The core cellular defense is the System Xc^-^–GSH–GPX4 axis. System Xc^-^ imports cystine for glutathione (GSH) synthesis. GSH is a essential cofactor for GPX4, which reduces cytotoxic lipid hydroperoxides (L-OOH) to harmless lipid alcohols (L-OH). Ferroptosis is promoted by the enzyme ACSL4, which esterifies free PUFAs (e.g., Arachidonic Acid - AA, Adrenic Acid - ADA) into membrane phospholipids (PUFA-PL), making them substrates for lipoxygenases (LOXs) and subsequent peroxidation. Cellular iron uptake via transferrin (Tf)/transferrin receptor 1 (TfR1) and subsequent release from ferritin via ferritinophagy (mediated by NCOA4) fuel the Fenton reaction, generating reactive oxygen species (ROS) that initiate and propagate lipid peroxidation. The transcription factor Nrf2 plays a dual role by activating antioxidant genes (e.g., SLC7A11, FTH1, FTL) but can promote iron release and ferroptosis under chronic activation via HO-1. Created with BioRender.

The primary defense against ferroptosis is the GSH-GPX4 axis. GPX4, using GSH as a cofactor, reduces cytotoxic lipid hydroperoxides (L-OOH) to benign lipid alcohols (L-OH) (13, 14). Cystine, the substrate for GSH synthesis, is imported by the system Xc^-^ transporter (a heterodimer of SLC7A11 and SLC3A2) (15, 16). In asthmatic airways, allergens like house dust mite can downregulate GPX4 and SLC7A11, crippling this defense and sensitizing cells to ferroptosis (17). A parallel, GSH-independent pathway is mediated by ferroptosis suppressor protein 1 (FSP1), which regenerates ubiquinol (CoQH_2_), a potent lipophilic antioxidant that halts lipid peroxidation chain reactions (13). The relative contribution of GPX4 and FSP1 in different lung cell types remain under investigation (18–20) (**Figure 2**).

#### 2.1.1 Pro-ferroptotic drivers: ACSL4 and iron

Acyl-CoA synthetase long-chain family member 4 (ACSL4) is key determinant of ferroptosis sensitivity. It catalyzes the esterification of long-chain polyunsaturated fatty acids (PUFAs, (e.g., arachidonic acid) into membrane phospholipids, making them substrates for peroxidation by enzymes like lipoxygenases (ALOXs) (21–23). High ACSL4 expression in asthmatic airway epithelium is a pro-ferroptotic hallmark (24). Iron, particularly labile Fe^2+^, is an essential catalyst for ferroptosis via the Fenton reaction. Cellular iron uptake (via transferrin receptor TFRC), storage (in ferritin), and release (via ferritinophagy mediated by NCOA4) are tightly regulated (25). In asthma, inflammatory cytokines can disrupt this homeostasis, leading to intracellular iron accumulation and heightened ferroptotic susceptibility (24).

#### 2.1.2 Transcriptional control by Nrf2

Nuclear factor erythroid 2-related factor 2 (Nrf2) is a master transcriptional regulator of the antioxidant response and a central mediator of cellular defenses against ferroptosis (26). Under basal conditions, Nrf2 is bound by the Keap1-Cul3 E3 ubiquitin ligase complex, leading to ubiquitination and proteasomal degradation (27). Upon oxidative stress, Keap1cysteine residues are modified, impairing Nrf2 degradation. Stabilized Nrf2 translocate to the nucleus, dimerizes with small Maf proteins, and binds to antioxidant response elements (AREs) in promoter regions of over 250 cytoprotective genes (28).

Nrf2 activation upregulates key ferroptosis defense components (29). It enhances expression of system Xc^-^ subunits (SLC7A11 and SLC3A2), promoting cystine uptake and GSH biosynthesis (30). Nrf2 also induces GPX4 expression, enabling the reduction of cytotoxic lipid hydroperoxides to harmless alcohols (31). Beyond these core elements, Nrf2 regulates a broad antioxidant network, including heme oxygenase-1 (HO-1, involved in iron recycling), NAD(P)H quinone dehydrogenase 1 (NQO1, which maintains antioxidant reduction), and enzymes involved in GSH synthesis (GCLC, GCLM) and regeneration (GSR) (32). Nrf2 also modulates iron metabolism, further influencing ferroptotic sensitivity (33). It promotes ferritin (FTH1 and FTL) expression, facilitating iron sequestration and preventing Fenton reactions (34). Additionally, Nrf2 can regulate ferroportin (FPN) expression to enhance iron export and modulate genes involved in heme and iron-sulfur cluster metabolism, contributing to iron homeostasis (35).

However, in the context of chronic diseases like asthma, sustained or dysregulated Nrf2 activation can exhibit paradoxical, pro-ferroptotic effects (36). For example, in asthmatic macrophages, persistent Nrf2 signaling increases HO-1 expression, releasing free iron from heme. If ferritin storage capacity is exceeded, this expanded the labile iron pool (LIP), predisposing cells to ferroptosis (37). This dual role is particularly evident in contexts of chronic, high-level oxidative stress, such as persistent allergen exposure in severe asthma, and in specific pro-inflammatory immune cells like M1 macrophages, where continuous heme degradation via HO-1 can overwhelm ferritin’s iron-buffering capacity. Thus, Nrf2 can function both as a protector against oxidative stress and an inadvertent promoter of ferroptosis under chronic activation conditions (38). This dual role complicates its therapeutic targeting in asthma, necessitating precise temporal and contextual modulation.

### 2.2 Regulation of iron metabolism: IRP2, ferritinophagy, and systemic influences

Iron availability, particularly labile Fe^2+^, is indispensable for lipid peroxidation and the execution of ferroptosis (39). Cellular iron levels are tightly controlled via the Iron Regulatory Protein/Iron Responsive Element (IRP/IRE) system, with IRP2 as the primary post-transcriptional regulator in many cell types (40). Under high iron conditions, stabilized IRP2 binds to IRE stem-loop structures in target mRNAs, modulating their stability and translation. Under high iron conditions, IRP2 is degraded, relieving its repression of ferritin/FPN translation and destabilizing TfR1 mRNA (41, 42).

Key regulatory targets include: Transferrin Receptor 1 (TfR1), whose mRNA is stabilized upon IRP2 binding to 3′ UTR IREs to enhance iron uptake; ferritin heavy and light chains (FTH1/FTL), whose translation is inhibited via 5′ UTR IREs to limit iron sequestration; and ferroportin (FPN), whose expression is suppressed to reduce iron export (43). In asthma, allergic inflammation disrupts this delicate balance, promoting IRP2 hyperactivation and aberrant iron accumulation in airway epithelial cells, thereby increasing ferroptotic susceptibility (44).

Ferritinophagy, a selective autophagic process mediated by the cargo receptor NCOA4, plays a pivotal role in iron release by targeting ferritin for lysosomal degradation (45). While physiologically important for iron recycling, dysregulated ferritinophagy—often triggered by oxidative stress or cytokines like IL-6 in asthma—floods the cytosol with iron, accelerating lipid peroxidation and ferroptosis in bronchial epithelial cells (46). This pathway represents a critical intersection between autophagy and iron metabolism in determining ferroptotic sensitivity.

Additional systemic and local factors further modulate iron homeostasis. The hormone hepcidin, elevated in chronic inflammation, binds to ferroportin inducing its internalization and degradation, thereby trapping iron within cells such as macrophages and potentiating pro-ferroptotic microenvironments (47). Moreover, iron-sulfur cluster (ISC) biogenesis not only supports electron transport but also modulates IRP2 activity; impaired ISC assembly can stabilize IRP2 and promote cellular iron overload (48).

In summary, ferroptosis is profoundly influenced by iron regulatory mechanisms centered on IRP/IRE signaling and ferritinophagy. In asthma, dysregulation of these pathways—through chronic IRP2 activation, excessive NCOA4-mediated ferritin degradation, or hepcidin-induced iron retention—fosters an iron-rich milieu in the airways that drives ferroptosis, amplifies epithelial injury, and exacerbates immune dysfunction. Therapeutic strategies targeting these iron regulatory nodes may inhibit ferroptosis without significantly disrupting systemic iron balance.

## 3 Organelle crosstalk in ferroptosis: how mitochondria-ER-lysosome axis drives asthma

**Figure 3:**
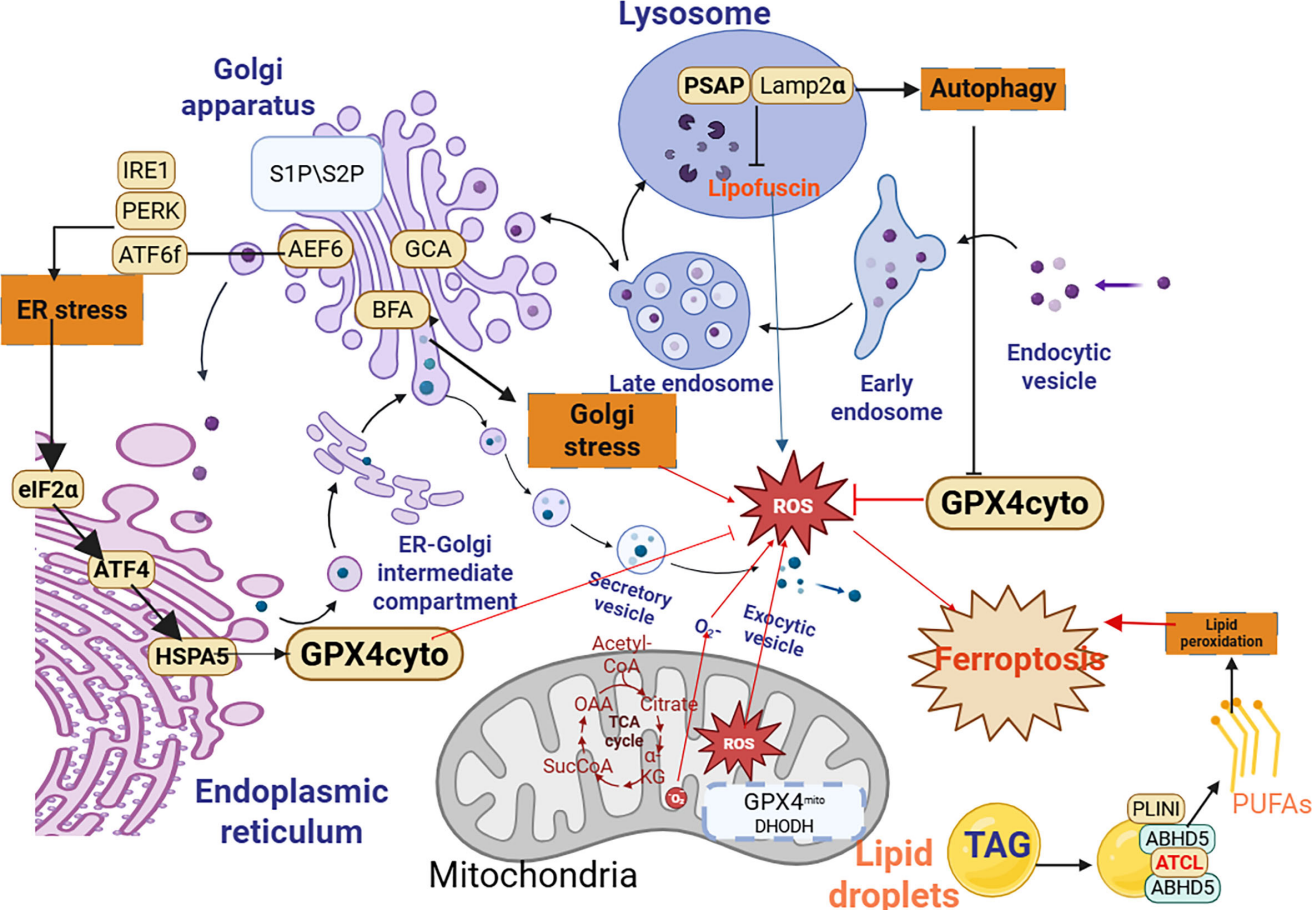
Organelle-specific regulation of ferroptosis in the asthmatic airway. Multiple organelles contribute to ferroptosis susceptibility through specialized mechanisms. Mitochondria: Allergens trigger DRP1-mediated fission, elevating ROS production and lipid peroxidation. Endoplasmic Reticulum (ER): ER stress and the unfolded protein response (UPR) upregulate lipid synthesis and disrupt calcium (Ca^2+^) homeostasis, promoting oxidative stress. Lysosomes: Ferritinophagy, mediated by NCOA4, degrades ferritin to release Fe^2+^, fueling Fenton reactions. Lysosomal membrane permeabilization (LMP) releases cathepsins and iron. Lipid Droplets (LDs): These store neutral lipids; lipophagy-mediated release of oxidation-sensitive PUFAs supplies substrates for peroxidation. Peroxisomes: Dysfunction impairs H_2_O_2_ catabolism (via catalase, CAT), elevating oxidative stress. Golgi Apparatus: Altered vesicular trafficking affects the localization of key enzymes and transporters (e.g., System Xc^-^). In asthma, environmental triggers dysregulate these organellar functions in airway epithelial and immune cells, fostering a pro-ferroptotic microenvironment that exacerbates inflammation and tissue injury.

Organelles do not act in isolation—their coordinated dysfunction drives ferroptosis in asthma. The regulation of ferroptosis involves a coordinated interplay among cellular organelles that modulate iron homeostasis, lipid peroxidation, and redox balance (49). Mitochondria, endoplasmic reticulum (ER), lysosomes, lipid droplets, peroxisomes, and the Golgi apparatus each contribute uniquely to this iron-dependent cell death process (19) (**Figure 3**). Central to this network is the mitochondria-ER-lysosome axis (MELA), which integrates metabolic and redox signals to amplify ferroptosis, rendering it a critical target for therapeutic intervention.

### 3.1 Mitochondria: the initiation hub of ferroptosis

Mitochondria serve as central regulators of metabolism and cell death, including ferroptosis (50). These organelles maintain redox balance and iron homeostasis; their dysfunction can elevate oxidative stress, initiating ferroptotic cell death (51, 52). Through oxidative phosphorylation (OXPHOS), mitochondria generate ATP but also produce ROS as byproducts (53). Under physiological conditions, antioxidant systems balance ROS production, but under stress, excess ROS accumulation can occur (54, 55), triggering lipid peroxidation of PUFAs in cellular membranes—a key event in ferroptosis (56).

Iron is vital for mitochondrial function, serving as a cofactor for proteins in the electron transport chain (ETC) and metabolic pathways. Mitochondria regulate iron storage and metabolism; dysregulation can lead to iron overload, catalyzing Fenton reactions and promoting lipid peroxidation (57). Mitochondrial ferritin sequesters excess iron, offering protection against oxidative damage (58). For example, mitochondrial ferritin overexpression attenuates cerebral ischemia/reperfusion injury by inhibiting ferroptosis (59). However, iron release under pathological conditions can exacerbate oxidative damage and drive ferroptosis (60).

Mitochondrial dynamics—fission and fusion—also influence ferroptotic susceptibility. Fission fragments mitochondria, increasing their vulnerability to damage and promoting the release of pro-death factors (61). Fusion maintains mitochondrial integrity and function, counteracting oxidative stress (18). Dysregulation shifts the balance toward cell death, including ferroptosis. Inhibited fusion enhances sensitivity to oxidative stress and ferroptosis.

Critically, mitochondria interact with other organelles, such as lysosomes and the ER, to maintain cellular homeostasis. Mitochondrial dysfunction destabilizes lysosomes, amplifying oxidative stress and ferroptotic signaling (62). The ER, involved in lipid synthesis, can affect lipid metabolism and ferroptotic sensitivity when dysfunctional (63). Mitophagy, the selective removal of damaged mitochondria, helps prevent ROS accumulation; its impairment leads to dysfunctional mitochondria accumulation, increasing oxidative damage and ferroptosis (64). In asthma, allergen-induced mitochondrial fission in airway epithelial cells enhances lipid peroxidation, underscoring the role of mitochondrial dysregulation in disease pathogenesis.

Recent studies highlight the role of mitochondria-ER contact sites (MAMs) in asthma-related ferroptosis. Allergen exposure promotes DRP1-mediated mitochondrial fission, increasing ROS generation. Through MAMs, Ca^2+^ is shuttled to the ER, activating the UPR pathway and upregulating ACSL4, thereby enhancing lipid peroxidation and ferroptosis (65). This organelle coupling provides a mechanistic basis for heightened ferroptotic sensitivity in asthmatic airways.

### 3.2 Endoplasmic reticulum: a regulator of lipid synthesis and calcium homeostasis

The ER is essential for protein synthesis, lipid metabolism, and calcium storage (66). Recent evidence highlights its role in regulating ferroptosis through stress responses, lipid biosynthesis, iron metabolism, and organelle interactions (67). As the primary site of lipid biosynthesis, the ER produces phospholipids and cholesterol vital for membrane integrity. ER stress can escalate lipid peroxidation, activating ferroptotic pathways. The ER also maintains redox homeostasis, crucial for ferroptosis regulation (68).

In response to oxidative stress or protein misfolding, the ER activates the unfolded protein response (UPR) to restore homeostasis. Persistent ER stress can promote apoptosis or ferroptosis by increasing oxidative stress and lipid peroxide accumulation (65). Specific UPR pathways upregulate lipid metabolism enzymes, facilitating peroxidation. The ER also houses system Xc^-^, regulating cysteine availability and GSH synthesis (69).

The ER’s role in lipid synthesis directly impacts ferroptotic susceptibility. PUFAs, synthesized in the ER, are more prone to peroxidation than saturated fatty acids (70). The ER also participates in ferritin synthesis and modification, influencing intracellular iron availability. Dysregulated lipid and iron metabolism in the ER can increase free iron levels, enhancing oxidative stress and lipid peroxidation (71).

ER-mitochondria interactions are particularly important for cellular resilience against ferroptosis. Mitochondrial dysfunction and ROS production can damage the ER, triggering further stress responses (72). Calcium signaling between the ER and mitochondria also influences cell survival; excessive calcium release can induce mitochondrial stress and cell death (73). In asthmatic airways, chronic ER stress may increase ferroptotic sensitivity, contributing to epithelial barrier dysfunction (69). Given its multifaceted role, the ER is a promising therapeutic target for ferroptosis-related diseases. Strategies alleviating ER stress, such as chemical chaperones or antioxidants, may inhibit ferroptosis (74). Modulating ER lipid composition toward less oxidizable lipids could also reduce ferroptotic susceptibility (75). The complex interplay between mitochondria and the ER underscores the integrated regulation of ferroptosis across organelles, highlighting the need for organelle-specific therapeutic strategies.

### 3.3 Lysosomal: iron release and autophagic regulation

Lysosomes are key organelles for degradation, recycling, and metabolic regulation (76). Their role in ferroptosis involves membrane integrity, iron metabolism, and interactions with other organelles (77). Lysosomal membrane stability is crucial for cellular homeostasis; oxidative stress can disrupt lysosomes, releasing hydrolytic enzymes and iron into the cytosol, amplifying oxidative stress and ferroptosis (66, 78). Lysosomes also facilitate autophagy—including chaperone-mediated autophagy (CMA) and macroautophagy—degrading damaged organelles and proteins. Reduced autophagic flux can lead to dysfunctional organelle accumulation, increasing oxidative stress and ferroptotic sensitivity (79).

Lysosomes are central to iron metabolism through ferritin degradation (80). Iron released via ferritinophagy can increase ROS generation via Fenton reactions if misregulated (81). In ferroptosis, lysosomal iron release catalyzes lipid peroxidation, overwhelming antioxidant defenses (39). Understanding lysosomal iron handling provides insights into ferroptosis regulation. Lysosomes interact with mitochondria and the ER. Mitochondrial dysfunction can induce lysosomal biogenesis and activation (82), while lysosomal impairment compromises mitochondrial health, potentially precipitating ferroptosis (83). Lysosomal destabilization can activate the NLRP3 inflammasome, triggering inflammation and cell death (84). Lysosomes and ER communicate through membrane contact sites, facilitating lipid transfer; disruption can disturb lipid homeostasis, increasing ferroptotic susceptibility (85). Targeting lysosomal function offers novel therapeutic avenues for ferroptosis-related diseases. Stabilizing lysosomal membranes or enhancing autophagic flux could mitigate ferroptosis in neurodegeneration or cancer (86). In asthma, lysosomal-mediated iron release may aggravate oxidative injury in bronchial epithelial cells, suggesting a mechanism for persistent inflammation (73).

### 3.4 Lipid droplets: lipid storage and release in axis fueling

Lipid droplets (LDs) are dynamic organelles storing neutral lipids, playing key roles in energy homeostasis and lipid metabolism (87). Their involvement in ferroptosis centers on lipid storage and release (88). LDs store PUFAs, which are highly susceptible to oxidation; PUFA release can increase lipid peroxide levels, promoting ferroptosis (88). Conversely, LDs can store saturated fatty acids, which are less oxidizable, potentially protecting against ferroptosis (89). The lipid composition of LDs is a critical determinant of ferroptotic susceptibility.

LDs also modulate cellular redox status by sequestering ROS and providing a controlled environment for lipid peroxidation (90). Under stress, LDs may store toxic lipids that, upon mobilization, trigger oxidative stress and ferroptosis (91). Lipophagy—selective LD degradation—can release free fatty acids, accelerating peroxidation and ferroptosis when iron levels or oxidative stress are high (92). In asthma, altered lipid metabolism may affect LD dynamics in alveolar macrophages, influencing inflammation and ferroptosis (93).

Notably, LDs cooperate with peroxisomes to regulate PUFA metabolism. PUFAs released from LDs require peroxisomal β-oxidation for metabolic processing. Impaired peroxisomal function leads to PUFA accumulation, enhancing lipid peroxidation and ferroptotic cell death. Clinical data indicate reduced expression of peroxisomal markers such as catalase (CAT) in lung tissues of asthma patients, underscoring the pathophysiological relevance of LD-peroxisome crosstalk in asthma-related ferroptosis.

### 3.5 Peroxisomes: lipid metabolism and ROS detoxification in axis balance

Peroxisomes are involved in lipid metabolism (e.g., PUFA β-oxidation) and ROS detoxification (e.g., hydrogen peroxide [H_2_O_2_] catabolism)—functions that directly modulate the ferroptosis-immune-metabolic axis (94). Their role in ferroptosis centers on regulating PUFA availability and oxidative stress, making them critical for axis homeostasis (95).

Peroxisomal β-oxidation generates lipid-derived signaling molecules (e.g., prostanoids) and reduces PUFA accumulation—limiting substrates for lipid peroxidation (96). Imbalanced fatty acid metabolism (e.g., reduced peroxisomal β-oxidation in asthma) leads to PUFA buildup, increasing peroxidation risk and ferroptosis (52). In asthmatic airway epithelial cells, allergen-induced downregulation of ACOX1—a key peroxisomal β-oxidation enzyme—markedly elevates intracellular PUFA levels (97). This metabolic shift enhances ferroptotic susceptibility by increasing the unsaturation of membrane phospholipids. Concurrently, the accumulated PUFAs serve as abundant precursors for pro-inflammatory eicosanoids, such as leukotriene B4 (LTB4), which recruits and activates neutrophils, thereby directly reinforcing the immune arm of the pathogenic axis.

Peroxisomes also degrade H_2_O_2_ via CAT, a process critical for reducing oxidative stress and limiting ferroptosis (98). Impaired peroxisomal function (e.g., reduced CAT activity) increases H_2_O_2_ accumulation, promoting Fenton reactions and lipid peroxidation (99, 100). In patients with severe asthma, bronchial epithelial cells exhibit diminished catalase (CAT) activity, which correlates with elevated systemic markers of lipid peroxidation (101). This association functionally links impaired peroxisomal ROS detoxification to the activation of the ferroptosis-immune-metabolic axis.

The interplay between iron and peroxisomal ROS is particularly relevant for axis progression. Iron overload (a hallmark of asthma) enhances ROS production via Fenton reactions (102); excess ROS, in turn, damages peroxisomal membranes, further impairing their metabolic and detoxification functions. This reciprocal positive feedback loop—where iron overload induces peroxisomal dysfunction, which in turn augments ROS production, and ultimately amplifies ferroptosis—sustains activation of the ferroptosis-immune-metabolic axis. Experimental and clinical evidence underscores the critical role of peroxisomal dysfunction in asthma (44). Iron chelation has been shown to ameliorate peroxisomal defects and suppress ferroptosis in asthmatic epithelial cells, validating the iron-peroxisome-ferroptosis loop as a therapeutic target (39). Furthermore, a key feature of the disease is the disrupted crosstalk between mitochondria and peroxisomes. While mitochondrial stress normally signals for peroxisomal biogenesis, this adaptive pathway is exhausted in chronic asthma, leading to peroxisomal depletion and a subsequent failure in redox and lipid homeostasis (103). Importantly, therapeutic strategies aimed at concurrently enhancing peroxisomal function and mitigating mitochondrial oxidative stress have demonstrated efficacy in ameliorating ferroptosis and improving lung function in experimental models, highlighting the promise of targeting this organellar network.

In asthma, peroxisomal dysfunction may impair antioxidant defenses in airway epithelium, increasing vulnerability to environmental triggers (e.g., air pollutants) (104). Clinical studies support this: exposure to diesel exhaust particles (DEPs) reduces peroxisomal ACOX1 expression in bronchial epithelial cells from asthma patients (105), exacerbating PUFA accumulation and ferroptosis. This suggests that peroxisomes act as a “sentinel” against environmental stress, and their dysfunction contributes to axis hyperactivation in polluted environments.

### 3.6 Golgi apparatus: lipid modification and protein trafficking in axis regulation

The Golgi apparatus modifies, sorts, and traffics proteins and lipids—functions that indirectly regulate the ferroptosis-immune-metabolic axis by influencing membrane composition and antioxidant defense (106). Its role in ferroptosis involves lipid modification, enzyme transport, and stress signaling—all of which impact axis progression (25).

The Golgi influences membrane lipid composition by modifying phospholipids (e.g., adding choline or ethanolamine headgroups) (107). These modifications affect membrane fluidity and susceptibility to oxidative damage: Golgi-derived phosphatidylcholine (PC) saturated fatty acids, reducing lipid peroxidation risk (108). In asthma, allergen-induced Golgi dysfunction perturbs its role in lipid metabolism, shifting membrane composition toward a higher abundance of peroxidation-sensitive, PUFA-containing phospholipids. This pro-ferroptotic lipid landscape facilitates membrane peroxidation, leading to cell death and the release of damage-associated molecular patterns (DAMPs). Subsequent immune cell activation by these DAMPs directly integrates Golgi-mediated lipid remodeling into the activation of the broader ferroptosis-immune-metabolic axis.

The Golgi also transports lipid metabolism enzymes (e.g., ACSL4, LOXs) to their target organelles (e.g., ER, mitochondria) (107). Dysregulated trafficking (e.g., reduced ACSL4 transport to the ER) modulates PUFA esterification and peroxidation risk (109). In asthmatic immune cells (e.g., Th17 cells), Golgi fragmentation (induced by IL-17A) impairs ACSL4 trafficking to the ER, leading to cytosolic ACSL4 accumulation (110). This mislocalization increases cytosolic PUFA esterification, generating lipid peroxides that promote ferroptosis in neighboring epithelial cells, reinforcing the immune-epithelial crosstalk within the axis.

Golgi stress (e.g., misfolded protein accumulation) can also promote ferroptosis by increasing oxidative stress. In asthma, allergen-induced protein misfolding (e.g., ovalbumin aggregates) triggers Golgi stress, activating the Golgi stress response (GSR) (111) Persistent Golgi stress response (GSR) upregulates NADPH oxidase 4 (NOX4) within the Golgi apparatus, leading to a substantial increase in localized ROS production. This Golgi-derived ROS pool subsequently exacerbates lipid peroxidation and drives ferroptotic cell death, which in turn compromises epithelial barrier integrity.

In asthma, dysregulated vesicular trafficking from the Golgi alters nutrient transporter expression in immune cells. Golgi dysfunction further contributes to axis activation by impairing the trafficking of key nutrient transporters. For instance, in M1 macrophages, disrupted Golgi function compromises system Xc^-^ delivery to the plasma membrane, thereby limiting cystine uptake, depleting glutathione, and sensitizing cells to ferroptosis (112). The ensuing death of these macrophages releases pro-inflammatory cytokines that amplify the inflammatory cycle. This mechanistic insight positions Golgi function as a novel therapeutic target. Compounds designed to stabilize Golgi integrity, such as brefeldin A analogs, have been shown to restore normal lipid metabolism and trafficking in experimental models (89). Preclinical studies corroborate that Golgi stabilization effectively reduces pro-ferroptotic membrane lipid composition, suppresses ferroptosis, and alleviates airway inflammation, validating this organelle as a promising target for therapeutic intervention.

## 4 Ferroptosis-immune-metabolic axis: a driver of asthma pathogenesis

The axis operates as a self-amplifying cycle that drives epithelial injury, inflammation, and remodeling. We dissect its three key stages: initiation by epithelial ferroptosis, amplification by immune cells, and metabolic sustenance (**Figure 1**).

### 4.1 Epithelial barrier dysfunction: the initiation of axis activation

The airway epithelium is the first line of defense against environmental triggers and the initial site of axis activation. In asthma, ferroptosis disrupts epithelial integrity through multiple interconnected mechanisms, initiating the axis cycle. A central event is the degradation of E-cadherin, a key adherens junction protein, resulting in loss of barrier cohesion and increased permeability (90).

This breach facilitates deeper penetration of allergens and pollutants into the submucosa, triggering innate and adaptive immune activation. Estrogen receptor α (ERα) has been identified as a mediator promoting ferroptosis in bronchial epithelial cells; its inhibition attenuates ferroptotic death and suppresses epithelial-mesenchymal transition (EMT), a process implicated in airway remodeling (91). Furthermore, mitochondrial dysfunction induced by common allergens such as house dust mite, augments lipid peroxidation via dynamin-related protein 1 (DRP1)-mediated fission, accelerating ferroptosis (92). Subsequent epithelial damage releases DAMPs that perpetuate inflammatory signaling and tissue injury.

### 4.2 Immune cell crosstalk: amplifying the axis cycle

Immune cells play a dual role in asthma, both responding to and actively promoting ferroptosis, thereby fueling a cycle of inflammation and cell death.

Macrophages polarized to the pro-inflammatory M1 phenotype demonstrate iron overload and heightened lipid peroxidation, worsening oxidative stress and ferroptosis (93). These cells secrete pro-inflammatory cytokines (e.g., TNF-α, IL-6) and ROS, aggravating epithelial injury (113). Conversely, M2 macrophages exert anti-ferroptotic effects via IL-10 secretion, which downregulates ACSL4 expression and enhances ferroportin (FPN)-mediated iron export, as recently demonstrated in Cell Death Differ (2024). This mechanism helps mitigate lipid peroxidation and ferroptotic cell death.

Neutrophils, central to steroid-resistant asthma, promote ferroptosis partly through arachidonate 15-lipoxygenase (ALOX15), catalyzing the oxidation of membrane-bound arachidonic acid into lipid peroxides (114). Th17 cells exacerbate neutrophilic inflammation and oxidative stress via IL-17A, which transcriptionally represses myocyte enhancer factor 2C (MEF2C). Consequently, MEF2C knockdown experiments—evidenced by reduced protein expression in Western blot and loss of nuclear localization in immunofluorescence—result in decreased GPX4 levels and increased ferroptotic susceptibility in airway epithelial cells (114). Additionally, immune checkpoints modulate ferroptosis in a cell-specific manner. For example, PD-L1 binding in airway epithelial cells activates the PI3K/Akt pathway, leading to GPX4 upregulation and ferroptosis resistance. In T cells, however, PD-1 engagement inhibits cystine uptake and promotes ferroptosis, illustrating the dual role of immune checkpoint signaling in asthma.

Type 2 innate lymphoid cells (ILC2s) modulate ferroptosis via activating transcription factor 4 (ATF4), which regulates GPX4 expression (115). Regulatory T cells (Tregs) and M2 macrophages, which normally resolve inflammation and suppress ferroptosis, are functionally impaired in asthma, tilting the balance toward persistent cell death and chronic inflammation.

### 4.3 Metabolic rewiring: sustaining axis dysregulation

Metabolic alterations in asthma directly promote ferroptosis by disrupting iron and lipid homeostasis. Dysregulated iron metabolism is characterized by increased expression of iron retention genes (e.g., hepcidin [HAMP], ferritin heavy and light chains [FTH1/FTL]) and reduced iron export via ferroportin (FPN), leading to intracellular iron accumulation (39). Excess iron catalyzes Fenton reactions, generating ROS that initiate and propagate lipid peroxidation. Concurrently, lipid metabolism is reprogrammed to favor polyunsaturated fatty acid (PUFA) incorporation into membranes. Enzymes such as ACSL4 activate PUFAs (e.g., arachidonic acid), enhancing their esterification into phospholipids and increasing membrane sensitivity to peroxidation (116). Furthermore, reduced activity of fatty acid desaturase 1 (FADS1) alters PUFA composition, further increasing ferroptosis vulnerability (117). These shifts are compounded by impaired antioxidant defenses, including downregulation of GPX4 and system Xc_-_ which diminish glutathione synthesis and the capacity to neutralize lipid hydroperoxides.

### 4.4 DAMP release and inflammatory amplification

Ferroptotic cells release DAMPs that act as alarmins, activating innate and adaptive immunity and perpetuating inflammatory cycles. High-mobility group box 1 (HMGB1), released from damaged cells, binds to Toll-like receptor 4 (TLR4) on macrophages, triggering NF-κB activation and production of pro-inflammatory cytokines such as IL-1β and TNF-α (98). Interleukin-33 (IL-33), an epithelial-derived alarmin upregulated in asthma, directly inhibits GPX4 expression—simultaneously exacerbating ferroptosis and promoting type 2 inflammation (118). Lipid peroxidation products including malondialdehyde (MDA) and 4-hydroxynonenal (4-HNE) form adducts with cellular proteins, generating neo-epitopes that activate immune cells and enhance antigen presentation (101). Additionally, adenosine triphosphate (ATP) released from ferroptotic cells activates the NLRP3 inflammasome, leading to caspase-1 activation and maturation of IL-1β and IL-18 (119). These DAMPs not only perpetuate local inflammation but also recruit additional immune cells into the airways, driving chronicity and structural remodeling.

### 4.5 Synthesis and clinical perspective

The interplay between epithelial dysfunction, immune cell activation, metabolic rewiring, and DAMP release creates a self-sustaining cycle that underlies asthma pathogenesis. Ferroptosis acts as both a cause and consequence of inflammation, making it a promising therapeutic target. Biomarkers such as plasma MDA and BALF 4-HNE reflect ferroptosis severity and correlate with lung function decline. Emerging therapies targeting ferroptosis—including inhaled nanoparticles delivering GPX4 mRNA or siRNA against ERα/ACSL4—show promise in preclinical models by selectively inhibiting lipid peroxidation without systemic immunosuppression (11). Future studies should employ spatial transcriptomics and single-cell metabolomics to identify patient endotypes amenable to precision targeting of the ferroptosis-immune-metabolic axis.

## 5 Immune cells and ferroptosis in asthma

Immune cells are central contributors to the pathogenesis of asthma, but their roles extend beyond simple pro- versus anti-ferroptotic categorization. Single-cell RNA sequencing studies have revealed remarkable heterogeneity and plasticity within immune populations in the asthmatic lung. Macrophages, for instance, exist on a spectrum of activation states beyond the classical M1/M2 dichotomy, and tissue-resident versus monocyte-derived subsets may have distinct impacts on ferroptosis. Similarly, T cell responses are shaped by the tissue microenvironment, and their pro- or anti-ferroptotic functions can vary accordingly. This section details how specific immune cell subsets interact with ferroptosis pathways while acknowledging this complexity.

Immune cells are central contributors to the pathogenesis of asthma, mediating chronic inflammation, tissue injury, and airway remodeling through a complex bidirectional relationship with ferroptosis. This iron-dependent cell death process both influences and is shaped by immune responses, establishing a vicious cycle that exacerbates disease severity. The following sections detail how specific immune cell subsets either promote or mitigate ferroptosis in the asthmatic lung (**Table 1**).

**Table 1:** Ferroptosis-related molecules in asthma.

Gene/Protein	Cell type	Role in asthma	Reference
GPX4↓	Airway epithelial cells	Enhances lipid peroxidation and cell death	(120)
SLC7A11↓	Alveolar macrophages	Reduces GSH synthesis, increasing ferroptosis susceptibility	(89)
ACSL4↑	Airway epithelial cells	Promotes PUFA integration into membranes, driving peroxidation	(121)
IL-33↑	Epithelial cells	Activates ST2 signaling, promoting inflammation and ferroptosis	(122)
ALOX15↑	Neutrophils	Catalyzes arachnidonic acid oxidation, triggering ferroptosis	(123)
MEF2C↓	Airway epithelium	Downregulation increases susceptibility to ferroptosis	(124)

"↓" indicates downregulation or decreased activity."↑" indicates upregulation or increased activity.

### 5.1 Pro-inflammatory drivers of ferroptosis

Multiple immune cell populations enhance inflammation and tissue damage in asthma by exacerbating oxidative stress and increasing cellular susceptibility to ferroptosis (125).

#### 5.1.1 M1 macrophages

Upon encountering allergens or pollutants, macrophages often polarize toward a pro-inflammatory M1 phenotype. These cells display intrinsic metabolic alterations conducive to ferroptosis, including iron retention—marked by elevated ferritin (FTH1/FTL) and hepcidin (HAMP) expression alongside reduced ferroportin (FPN) levels (126). This iron overload fuels Fenton reactions and lipid peroxidation. Furthermore, M1 macrophages generate abundant ROS and secrete pro-inflammatory cytokines (e.g., TNF-α, IL-6), which propagate lipid peroxidation and impair antioxidant mechanisms in neighboring structural cells (127). Hyperactivation of NF-κB and suppression of peroxisome proliferator-activated receptor gamma (PPARγ) pathways further reinforce this pro-ferroptotic phenotype.

#### 5.1.2 Th17 cells

Th17 cells exacerbate neutrophilic inflammation and oxidative stress in asthmatic airways through the secretion of IL-17A and other cytokines (128). IL-17A promotes ferroptosis through multiple mechanisms: it transcriptionally represses myocyte enhancer factor 2C (MEF2C), a key positive regulator of GPX4 expression, thereby crippling the cellular antioxidant defense. Additionally, IL-17A synergizes with IFN-γ to inhibit system Xc^-^ activity, leading to glutathione depletion. Th17-derived cytokines also promote intracellular iron accumulation in structural lung cells, further accelerating ferroptotic damage. Recent studies (2023-2025) have demonstrated that IL-17A directly upregulates pro-ferroptotic mediators such as ACSL4 while suppressing protective factors like myocyte enhancer factor 2C (MEF2C) in airway epithelium, thereby creating a feed-forward loop of lipid peroxidation (129–131). Th17-derived cytokines also promote intracellular iron accumulation and glutathione depletion in structural lung cells, accelerating ferroptotic damage (132).

#### 5.1.3 Neutrophils

Neutrophils are key effectors in steroid-resistant asthma and intensify ferroptosis through several mechanisms (133). They produce large quantities of ROS and express ALOX15, which directly oxidizes membrane PUFAs. The release of neutrophil elastase and matrix metalloproteinases disrupts epithelial barrier integrity, permitting further oxidative insult (134). Additionally, neutrophil extracellular traps (NETs) contribute to iron dysregulation and lipid peroxidation, perpetuating epithelial injury and inflammatory amplification (135).

Pro-inflammatory immune cells (M1 macrophages, Th17 cells, neutrophils) promote ferroptosis via iron accumulation, lipid peroxidation, and ROS production. In contrast, anti-inflammatory cells (M2 macrophages, Tregs) suppress ferroptosis through iron export, antioxidant upregulation, and anti-cytokine release. Immune checkpoints such as PD-1/PD-L1 and TIM-3 further modulate ferroptosis in a cell-specific manner. Targeting these immune-ferroptosis interactions offers therapeutic potential for severe asthma.

### 5.2 Anti-inflammatory resolvers

Certain immune cell populations play a protective role by resolving inflammation and suppressing ferroptosis.

#### 5.2.1 M2 macrophages

M2 macrophages counteract ferroptosis by promoting iron export via ferroportin and enhancing antioxidant capacity through upregulation of GPX4 and glutathione synthesis (136). These cells also secrete anti-inflammatory cytokines (e.g., IL-10) that dampen M1 responses and facilitate tissue repair (137). In asthma, however, the balance is skewed toward M1 dominance, resulting in impaired clearance of damaged cells and unabated ferroptosis (138).

#### 5.2.2 Regulatory T cells (Tregs)

Tregs help maintain immune homeostasis and mitigate ferroptosis by suppressing Th17 and neutrophil responses. Through the secretion of IL-10 and transforming growth factor-beta (TGF-β), they reduce oxidative stress and lipid peroxidation. Tregs also promote the expression of ferroptosis suppressors such as GPX4 and ferroptosis suppressor protein 1 (FSP1) (139). Their functional impairment in asthmatic patients permits uncontrolled ferroptosis and contributes to disease progression.

### 5.3 Immune checkpoints

#### 5.3.1 PD-1/PD-L1 axis

The programmed cell death protein 1/programmed death-ligand 1 (PD-1/PD-L1) pathway is upregulated in asthma and exhibits context-dependent effects on ferroptosis (132). Although PD-1 activation generally suppresses T cell responses, it may promote ferroptosis in T cells by inhibiting cystine uptake and glutathione synthesis (140). In airway epithelial cells, by contrast, PD-L1 expression has been associated with enhanced resistance to ferroptosis (141). The net effect of this checkpoint signaling likely varies by cellular context and disease stage (142).

#### 5.3.2 TIM-3

TIM-3 is linked to T cell exhaustion and defective immune regulation in asthma (143). Emerging evidence indicates that TIM-3 can promote ferroptosis in T cells by suppressing AMP-activated protein kinase (AMPK) signaling and enhancing lipid peroxidation (144). I In macrophages, TIM-3 signaling alters iron metabolism, further promoting oxidative stress. Therapeutic inhibition of TIM-3 may therefore restore immune homeostasis and reduce ferroptosis in severe asthma (145).

In summary, immune cells significantly influence ferroptosis in asthma through the dysregulation of iron metabolism, promotion of lipid peroxidation, and impairment of antioxidant defenses. Therapeutic strategies that target immune-ferroptosis crosstalk—via macrophage polarization reprogramming, immune checkpoint modulation, or specific ferroptosis inhibitors—hold promise for patients with severe or steroid-resistant asthma.

## 6 The ferroptosis-immune-metabolic axis in asthma

**Figure 4:**
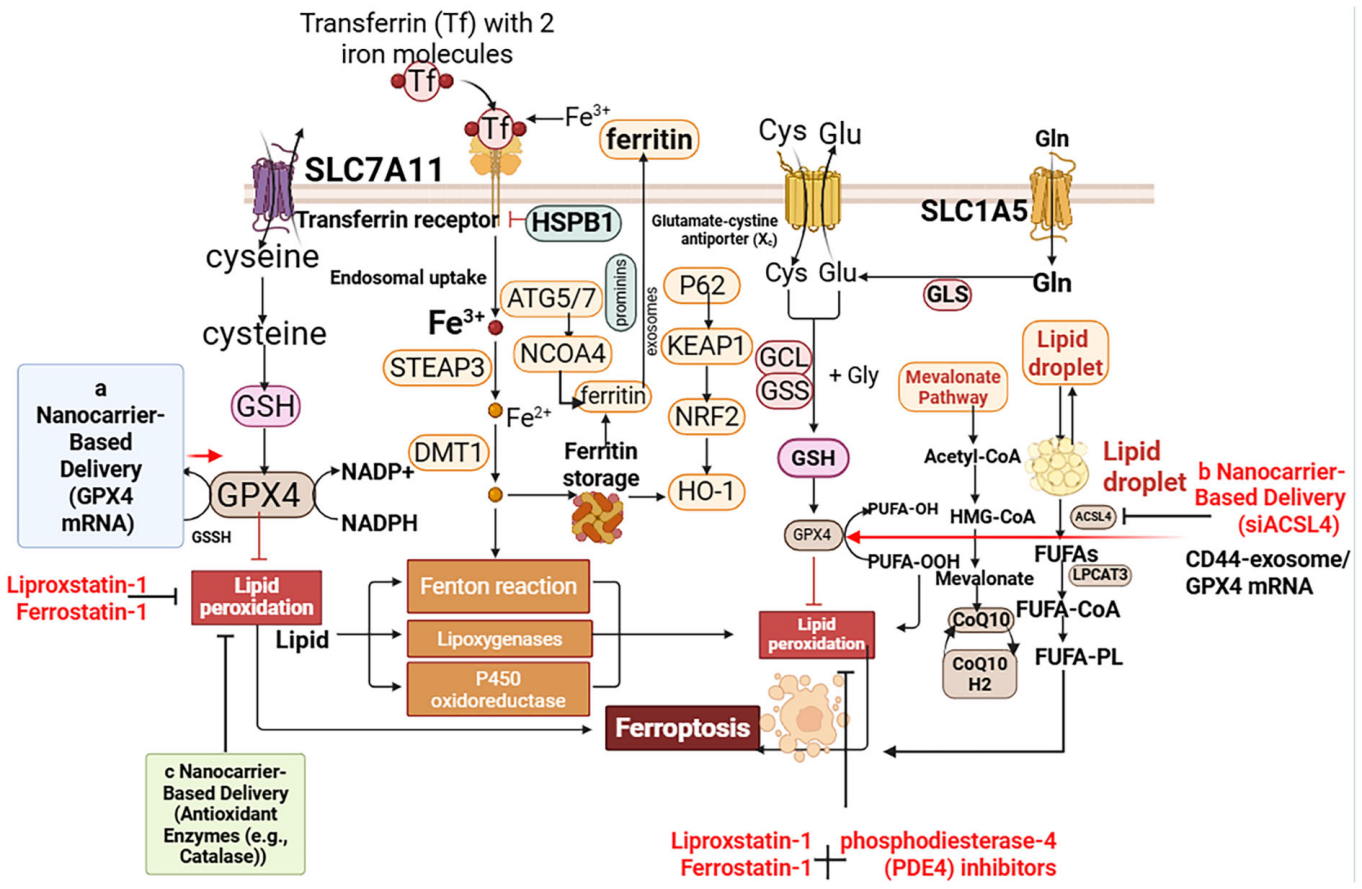
Emerging therapeutic strategies targeting ferroptosis in asthma. Novel precision medicine approaches aim to disrupt the ferroptosis-immune axis. 1. Inhaled Ferroptosis Inhibitors: Small molecule inhibitors like Liproxstatin-1 and Ferrostatin-1 can be nebulized to directly scavenge lipid radicals in the airways. 2. Nanocarrier-Based Delivery: Inhaled nanoparticles (e.g., lipid nanoparticles, mesenchymal stem cell (MSC)-derived exosomes) can be engineered for targeted delivery. They can carry: **(a)** GPX4 mRNA to restore the primary antioxidant defense in epithelial cells or macrophages. **(b)** siRNA to knock down pro-ferroptotic genes like ACSL4 or ERα. **(c)** Antioxidant Enzymes (e.g., Catalase). 3. Combination Therapies: Ferroptosis inhibitors show synergy with existing therapies (e.g., corticosteroids) to overcome steroid resistance, particularly in neutrophilic asthma endotypes. 4. Biomarker-Guided Therapy: Stratifying patients based on ferroptosis biomarkers (e.g., plasma MDA, BALF 4-HNE, genetic polymorphisms in FADS1) enables personalized treatment decisions. These strategies offer the potential for lung-specific intervention without systemic immunosuppression. Created with BioRender (Created in https://BioRender.com).

The chronic inflammation and structural remodeling that characterize asthma are sustained through a dynamic interplay between ferroptosis, immune activation, and metabolic reprogramming. This triad forms a pathogenic axis that drives disease progression, particularly in severe and treatment-resistant cases, and presents novel opportunities for therapeutic intervention (**Figure 4**).

### 6.1 Ferroptosis as an inflammation amplifier

Ferroptosis functions as a potent amplifier of airway inflammation through the accumulation of lipid peroxidation products, particularly malondialdehyde (MDA) and 4-hydroxynonenal (4-HNE) (146). These bioactive aldehydes activate crucial inflammatory signaling hubs, including the NF-κB pathway and NLRP3 inflammasome, triggering the maturation and release of pro-inflammatory cytokines such as IL-1β and IL-18 (147). This signaling cascade recruits and activates both innate and adaptive immune cells, perpetuating a cycle of oxidative damage and inflammation. This process is self-reinforcing: inflammation-derived oxidative stress promotes further ferroptosis, while dying ferroptotic cells release DAMPs that activate surrounding structural and immune cells, intensifying the inflammatory response (7). This vicious cycle represents a fundamental mechanism underlying disease chronicity in asthma, contributing significantly to persistent tissue damage and remodeling. While this model is compelling, the causal relationships within this self-amplifying cycle are supported by varying levels of evidence. Some links, such as the release of DAMPs from ferroptotic cells and their activation of immune responses, are well-established. Others, particularly the precise mechanisms by which immune-derived signals feedback to directly initiate ferroptosis in specific cell types, represent strong logical inferences based on associative data and require further causal validation.

### 6.2 Classical and non-classical pathways of ferroptosis regulation

The regulation of ferroptosis in the asthmatic airway involves both canonical and alternative mechanisms that determine cellular susceptibility to this form of cell death.

#### 6.2.1 Classical pathways

The canonical pathway is typically initiated by allergen exposure (e.g., house dust mite, ovalbumin), which leads to the downregulation of key antioxidant defenses in airway epithelial cells (148). Central to this process is the suppression of GPX4 and the cystine/glutamate antiporter, system Xc^^-^^. This disruption severely compromises cellular capacity to reduce lipid hydroperoxides and synthesize glutathione, thereby dramatically increasing susceptibility to iron-dependent lipid peroxidation and ferroptotic cell death (148).

#### 6.2.2 Non-classical pathways

Beyond the canonical GPX4/system Xc^-^ axis, emerging research has revealed additional mechanisms that critically regulate ferroptotic sensitivity in asthma. These non-canonical pathways often operate independently of glutathione metabolism, adding layers of complexity to ferroptosis regulation (149). A prominent example involves long non-coding RNAs (lncRNAs), such as LINC01133, which demonstrates potent antiferroptotic effects within the airway epithelium (150). Its mechanism involves binding to and stabilizing the mRNA transcript of ferroptosis suppressor protein 1 (FSP1), a key component of a parallel antioxidant system that utilizes ubiquinone (CoQ10) to neutralize lipid peroxides, thereby establishing a crucial post-transcriptional regulatory checkpoint (151). Clinical studies have begun to reveal the relevance of this pathway, with LINC01133 expression showing significant downregulation in bronchial epithelial cells from patients with severe asthma, correlating with increased lipid peroxidation markers.

Hormonal signaling pathways also contribute significantly to this regulatory framework. Estrogen receptor α (ERα) activation has been identified as a promoter of ferroptotic cell death in bronchial epithelial cells, potentially through modulation of iron homeostasis and lipid metabolic genes, including transcriptional repression of GPX4 and enhanced expression of transferrin receptor 1 (TfR1) (152). Pharmacological inhibition of ERα signaling attenuates ferroptosis, providing mechanistic insight into the sexual dimorphism observed in asthma prevalence and severity, where premenopausal females often exhibit higher disease burden.

Environmental factors further modulate these non-canonical pathways. Exposure to pollutants such as dibutyl phthalate (DBP) can exacerbate ferroptotic processes. DBP and similar compounds disrupt the core Nrf2/HO-1 antioxidant signaling axis, a critical cellular defense program (153). This impairment cripples the adaptive stress response, effectively lowering the threshold for oxidative damage and rendering airway structural cells markedly more vulnerable to ferroptosis upon encounter with other environmental triggers. Other emerging non-canonical regulators include the mevalonate pathway, which generates coenzyme Q10 (CoQ10), an essential cofactor for FSP1. Statins, which inhibit HMG-CoA reductase, can indirectly promote ferroptosis by depleting CoQ10 levels, suggesting a potential mechanism for the rare pulmonary side effects of these drugs and highlighting another layer of metabolic regulation.

### 6.3 Metabolic-immune dialogue in ferroptosis

Metabolic reprogramming serves as a critical nexus linking immune function to ferroptotic susceptibility. Activated immune cells in the asthmatic microenvironment undergo profound metabolic shifts that directly influence redox balance. For instance, M1 macrophages and neutrophils exhibit enhanced glycolytic flux and pentose phosphate pathway activity, which can paradoxically increase ROS production and deplete antioxidant reserves (154). Conversely, the functional efficacy of anti-inflammatory resolvers, such as M2 macrophages and T regulatory cells, depends on oxidative phosphorylation and fatty acid oxidation—metabolic programs that support their antioxidant and repair functions (112). This metabolic dialogue extends to iron availability: pro-inflammatory signals alter the expression of iron import (e.g., transferrin receptor 1, TfR1) and storage (e.g., ferritin) proteins, creating an iron-rich environment that primes cells for ferroptosis. Thus, the immunometabolic landscape of the asthmatic airway directly dictates cellular fate by modulating the core executers of ferroptosis, integrating inflammatory cues with metabolic and iron homeostasis to drive disease progression.

## 7 Therapeutic implications and challenges

The identification of ferroptosis as a critical mechanism in asthma pathogenesis, especially in severe and treatment-resistant cases, has unveiled new opportunities for disease-modifying interventions. Moving beyond conventional symptomatic management, therapeutic targeting of this iron-dependent peroxidative cell death pathway represents a paradigm shift aimed at preserving epithelial integrity and restoring redox balance. This section evaluates the translational potential of ferroptosis modulation, discusses precision medicine approaches, and outlines key challenges for clinical application (**Table 2**).

**Table 2:** Emerging therapies targeting ferroptosis in asthma.

Therapy	Target	Stage	Outcome	Identifier
Liproxstatin-1 (nebulized)	Lipid ROS	Preclinical	Reduces airway inflammation	(155)
siRNA@MSN@CM (inhaled)	Epithelial ERα	Preclinical	Attenuates ferroptosis and EMT	(91)
Ferrostatin-1 + Corticosteroids	GPX4/Inflammation	Phase I/II	Improves lung function (interim)	(156)
CD44-exosome/GPX4 mRNA	Alveolar macrophages	Preclinical	80% reduction in BAL 4-HNE	(157)

### 7.1 Biomarkers for patient stratification

Accurate identification of patients with ferroptosis-prone asthma is essential for precision therapy. Compared to existing diagnostic methods like FeNO and sputum eosinophilia, ferroptosis-related biomarkers offer potential advantages in identifying novel endotypes, predicting steroid resistance, and reflecting distinct pathophysiological processes. However, they also face disadvantages including the invasiveness of BALF collection, lack of standardized assays for lipid peroxides, and ongoing validation requirements. Their primary utility lies not in initial diagnosis but as supplementary tools for precision medicine—stratifying existing asthma patients for targeted therapy, predicting exacerbation risk, and monitoring response to ferroptosis-directed treatments.

Biomarker profiles can be stratified by endotype:

Eosinophilic Asthma: Serum IL-33 (promotes ferroptosis) and fractional exhaled nitric oxide (FeNO, correlates positively with lipid peroxidation products).

Neutrophilic Asthma: BALF ALOX15 (neutrophil-derived) and plasma hepcidin (indicator of iron retention).

Clinical validity is supported by real-world evidence. Clinical evidence has established that elevated levels of the lipid peroxidation product 4-HNE in bronchoalveolar lavage fluid are significantly associated with poorer lung function in patients with neutrophilic asthma, underscoring its relevance as a biomarker for disease severity and potential treatment response (158).

Additional biomarker candidates include plasma MDA and BALF 4-HNE, which reflect oxidative damage and correlate with disease severity (159), and genetic markers such as FADS1 polymorphisms that influence membrane lipid unsaturation and peroxidation susceptibility. These biomarkers may guide the use of ferroptosis-directed therapies and tailored nutritional interventions with omega-3 fatty acids.

### 7.2 Targeted therapeutic strategies

#### 7.2.1 Eosinophilic asthma

This endotype is characterized by elevated type 2 inflammation and pronounced epithelial ferroptosis. A promising therapeutic approach involves using inhaled lipid nanoparticles to deliver GPX4 mRNA, aiming to directly augment antioxidant defenses in airway epithelial cells. Encouraging interim results from an ongoing clinical trial have reported clinically meaningful improvements in lung function, supporting the further development of this mRNA-based strategy (160).

#### 7.2.2 Neutrophilic asthma

Given the central role of pro-ferroptotic lipid metabolism in this asthma subtype, targeting arachidonate 15-lipoxygenase (ALOX15)—which is highly expressed in neutrophilic infiltrates—has emerged as a rational therapeutic strategy. This approach is supported by clinical evidence; for instance, a recent phase II trial demonstrated that combining an ALOX15 inhibitor with low-dose corticosteroids significantly reduced the annualized exacerbation rate compared to corticosteroid monotherapy (161).

#### 7.2.3 Corticosteroid resistance

A subset of patients exhibits glucocorticoid resistance due to chronic Nrf2 activation, which paradoxically enhances ferroptotic susceptibility. Preclinical models indicate that co-administration of the Nrf2 inhibitor Brusatol with corticosteroids reduces iron overload and lipid peroxidation. *In vitro*, this combination decreased ferroptosis in human bronchial epithelial cells by 52%, offering a novel strategy to restore steroid sensitivity.

#### 7.2.4 Broad-spectrum ferroptosis inhibitors

First-generation inhibitors such as liproxstatin-1 and ferrostatin-1 have shown efficacy across multiple preclinical asthma models. These agents mitigate airway hyperreactivity, inflammatory infiltration, and remodeling through potent lipid radical scavenging (162). By curbing membrane peroxidation, they help preserve epithelial barrier function and dampen downstream inflammation.

#### 7.2.5 Nanoparticle-mediated delivery

To improve lung-specific targeting and reduce systemic side effects, advanced inhaled nanocarriers—including functionalized lipid nanoparticles and extracellular vesicles—are in development (163). These systems allow targeted delivery of therapeutic nucleic acids, such as GPX4 mRNA to enhance antioxidant capacity or ACSL4-siRNA to limit peroxidation-prone lipid pools (164), enabling cell-type-specific suppression of ferroptosis.

#### 7.2.6 Combination therapies

Ferroptosis inhibitors exhibit synergistic effects with existing asthma drugs. They may reverse steroid resistance by countering oxidative inflammatory pathways and enhance the efficacy of phosphodiesterase-4 (PDE4) inhibitors (e.g., roflumilast), particularly in neutrophilic asthma (165). These multi-target strategies reflect a holistic approach to complex disease pathophysiology.

#### 7.2.7 Ferroptosis inhibitors

First-generation ferroptosis inhibitors such as liproxstatin-1 and ferrostatin-1 have demonstrated significant efficacy across multiple preclinical asthma models. These compounds attenuate key pathological features including airway hyperreactivity, inflammatory cell infiltration, and structural remodeling through their potent ability to scavenge lipid radicals (166). By halting membrane peroxidation chains, they prevent the loss of epithelial barrier function and subsequent inflammatory amplification.

#### 7.2.8 Nanoparticle-mediated delivery

To enhance lung-specific targeting while minimizing systemic exposure, advanced inhaled nanocarriers—including lipid nanoparticles and extracellular vesicles—are under development (167). These platforms enable targeted delivery of therapeutic nucleic acids, such as GPX4 mRNA to restore antioxidant capacity or ACSL4-siRNA to reduce substrate availability for lipid peroxidation (168). Such approaches offer unprecedented precision in suppressing ferroptosis within specific pulmonary cell populations.

#### 7.2.9 Combination therapies

Ferroptosis inhibitors demonstrate promising synergism with existing asthma therapeutics. They can potentially reverse steroid resistance by mitigating oxidative stress-mediated inflammatory pathways. Furthermore, their combination with phosphodiesterase-4 (PDE4) inhibitors (e.g., roflumilast) enhances anti-inflammatory efficacy, particularly in neutrophilic asthma phenotypes (169). These combination strategies represent a multifaceted approach to addressing the complex pathophysiology of severe asthma.

### 7.3 Ongoing challenges and future directions

The translation of ferroptosis-targeted therapies faces several significant challenges that require careful consideration:

#### 7.3.1 Nanocarrier delivery challenges

While nanocarriers offer promising targeting capabilities, they face substantial translational hurdles including potential immunogenicity, nebulizer compatibility and stability, batch-to-batch consistency in manufacturing, and difficulties achieving uniform deep lung penetration and cellular uptake across different airway regions and cell types.

#### 7.3.2 Biomarker standardization challenges

The practical implementation of ferroptosis biomarkers faces difficulties in standardizing measurements of reactive lipid species like MDA and 4-HNE across clinical laboratories, defining clinically validated cut-off values for therapeutic decision-making, and the invasive nature of obtaining optimal samples like BALF for routine monitoring.

#### 7.3.3 Safety and specificity concerns

Long-term safety assessment remains paramount, particularly regarding the theoretical risk of impairing tumor surveillance by chronically inhibiting a controlled cell death pathway, potential off-target effects of RNA-based therapies, and the challenge of achieving cell-type specificity without affecting essential ferroptosis functions in other tissues.

#### 7.3.4 Cell-type-specific targeting

A significant translational challenge lies in achieving selective drug delivery to relevant pulmonary cell populations (e.g., epithelial cells, specific immune subsets) without affecting off-target cells. Advanced nanodelivery systems functionalized with cell-specific ligands (e.g., anti-CD44 antibodies) are currently under investigation to enhance targeting precision and therapeutic efficacy.

#### 7.3.5 Phenotyping and endotyping

Emerging technologies such as single-cell RNA sequencing and spatial metabolomics promise to unravel the heterogeneity of ferroptosis activation across asthma endotypes. These approaches will enable identification of patient-specific ferroptotic signatures and facilitate the development of personalized therapeutic combinations tailored to individual molecular profiles.

#### 7.3.6 Clinical translation

Successful translation of ferroptosis-targeted therapies requires methodical progression through several key stages: validation of candidate biomarkers in well-characterized human cohorts, optimization of inhalation formulations for consistent pulmonary deposition, and demonstration of safety and efficacy through rigorously designed clinical trials. Overcoming these hurdles will be essential for harnessing the full potential of ferroptosis inhibition in severe asthma management.

## 8 Conclusion and future perspectives

The recognition of ferroptosis as a critical mechanistic link between dysregulated metabolism, immune activation, and chronic airway inflammation establishes the Ferroptosis-Immune-Metabolic Axis as a transformative conceptual framework for understanding asthma pathogenesis. This integrative perspective elucidates how iron-dependent lipid peroxidation not only initiates epithelial damage but also propagates inflammatory signaling and metabolic reprogramming, creating a self-reinforcing cycle of disease progression. Key findings underscoring this paradigm include the dual role of oxidative metabolites as both effectors and amplifiers of immunity, the substantial therapeutic potential of ferroptosis inhibition, and the emergence of peroxidation-related biomarkers for predictive patient stratification.

Despite the significant progress in unraveling the role of ferroptosis in asthma, several critical research gaps remain to be addressed. First, the functional role of ferroptosis in distinct asthma subtypes—specifically pediatric asthma and asthma-COPD overlap syndrome (ACOS)—has not been clearly defined. Current studies primarily focus on adult asthma populations, leaving uncertainties about whether ferroptosis exhibits subtype-specific mechanisms or regulatory patterns in these understudied groups. Second, while single-cell sequencing technologies have revealed heterogeneity in ferroptotic cells (e.g., differences across airway epithelial cell subtypes), the functional validation of these subtype-specific ferroptotic characteristics is lacking. This gap limits the translation of observational findings into mechanistic insights and targeted interventions.

To advance this conceptual framework and translate these insights into clinical practice, future research should prioritize the following strategic directions:

1. Spatial Mapping of Ferroptosis:

The application of spatial multi-omics platforms and advanced imaging technologies will be essential to elucidate cell- and zone-specific ferroptosis signatures within the asthmatic airway microenvironment. These approaches should clarify the spatial relationships between ferroptotic activity, immune infiltrates, and metabolic alterations, thereby refining our understanding of the proposed axis within its architectural context.

2. Genetic and Metabolic Targeting:

The development of cell-specific ferroptosis loss-of-function models (e.g., epithelial- or immune-specific GPX4 or ACSL4 knockout systems) will enable precise dissection of compartment-specific contributions to inflammation and metabolic dysregulation. These investigations will facilitate the design of targeted therapeutic interventions tailored to specific cellular mechanisms.

3. Combination Immuno-Ferroptosis Therapy:

There is a compelling need to evaluate rational drug combinations that simultaneously target ferroptosis and immune pathways. Strategic combinations of ferroptosis inhibitors with immunomodulators (e.g., anti-TIM-3, anti-IL-4Rα) may disrupt both oxidative injury and maladaptive immune responses, addressing the multi-axis nature of severe asthma. A promising priority lies in developing dual-target nanotherapeutics capable of concurrently suppressing ferroptosis drivers (e.g., ACSL4) and amplifying immunoregulatory pathways (e.g., Treg activation), thereby enabling synergistic correction of both redox and immune dysfunction.

4. Biomarker Validation and Clinical Translation:

The field must prioritize the validation of clinical biomarkers that accurately reflect ferroptosis activity (e.g., MDA, 4-HNE, extracellular ferritin). Concurrently, advancing inhaled nanotherapeutics for targeted pulmonary delivery will be crucial for enabling stratified medicine approaches and precision intervention in asthma management. Furthermore, constructing asthma digital twin models incorporating ferroptosis-related biomarkers may offer a powerful tool for clinical subtyping and personalized therapeutic simulation.

5. Development of Dual-Target “Ferroptosis-Immunity” Nanotherapeutics:

A high-priority direction involves the design and development of nanoscale drug delivery systems that simultaneously target ferroptosis and immune regulation. For instance, nanotherapeutics engineered to inhibit ACSL4 (a key mediator of ferroptosis) while activating regulatory T cells (Treg)—which modulate excessive immune responses—could synergistically address both oxidative stress and immune dysregulation, two core drivers of asthma progression. This dual-target strategy has the potential to overcome the limitations of single-mechanism therapies and improve therapeutic efficacy in severe asthma.

6. Establishment of Asthma Digital Twin Models Based on Ferroptosis Biomarkers.

Another priority is the construction of digital twin models for asthma that integrate ferroptosis-related biomarkers. These models, which simulate individual patient characteristics (e.g., ferroptosis activity, immune profile, metabolic status), could serve as powerful tools for clinical stratification. By incorporating real-time patient data, digital twins can predict disease progression, optimize treatment selection, and enable personalized intervention—ultimately advancing precision medicine in asthma care.

By anchoring further investigations within the Ferroptosis-Immune-Metabolic Axis framework, future studies can accelerate the development of mechanism-based therapies that simultaneously address oxidative stress, immune dysregulation, and metabolic dysfunction. This integrated approach holds significant promise for improving outcomes in patients with severe and refractory asthma who remain underserved by current therapeutic options. In conclusion, the ferroptosis-immune-metabolic axis represents a paradigm shift in asthma research—offering a unified framework to understand disease heterogeneity and develop precision therapies. Addressing remaining research gaps and prioritizing translational strategies will be critical to harnessing the full potential of this axis for improving patient care.

## Abbreviations

GPX4: Glutathione peroxidase 4
DAMPs: Damage-associated molecular patterns
MDA: Malondialdehyde
PUFA: Polyunsaturated fatty acid
GSH: Glutathione
L-OOH: Lipid hydroperoxides
L-OH: Lipid alcohols
AA: Arachidonic Acid
ADA: Adrenic Acid
PUFA-PL: PUFA-containing phospholipids
LOXs: Lipoxygenases
Tf: Transferrin
TfR1: Transferrin receptor 1
NCOA4: Nuclear receptor coactivator 4
ROS: Reactive oxygen species
Nrf2: Nuclear factor erythroid 2-related factor 2
SLC7A11: Solute carrier family 7 member 11
FTH1: Ferritin heavy chain 1
FTL: Ferritin light chain
HO-1: Heme oxygenase-1
ACSL4: Acyl-CoA synthetase long-chain family member 4
ER: Endoplasmic reticulum
UPR: Unfolded protein response
Ca²⁺: Calcium
LMP: Lysosomal membrane permeabilization
LDs: Lipid Droplets
CAT: Catalase
ERα: Estrogen receptor α
EMT: Epithelial-mesenchymal transition
DRP1: Dynamin-related protein 1
ALOX15: Arachidonate 15-lipoxygenase
ILC2s: Type 2 innate lymphoid cells
ATF4: Activating transcription factor 4
Th17: T helper 17 cell
MEF2C: Myocyte enhancer factor 2C
Tregs: Regulatory T cells
HAMP: Hepcidin
FPN: Ferroportin
FADS1: Fatty acid desaturase 1
HMGB1: High-mobility group box 1
TLR4: Toll-like receptor 4
IL-1β: Interleukin-1 beta
TNF-α: Tumor necrosis factor-alpha
IL-33: Interleukin-33
4-HNE: 4-Hydroxynonenal
ATP: Adenosine triphosphate
NLRP3: NLR family pyrin domain containing 3
IL-18: Interleukin-18
BALF: Bronchoalveolar lavage fluid
siRNA: Small interfering RNA
PPARγ: Peroxisome proliferator-activated receptor gamma
IFN-γ: Interferon-gamma
NETs: Neutrophil extracellular traps
FSP1: Ferroptosis suppressor protein 1
PD-1: Programmed cell death protein 1
PD-L1: Programmed death-ligand 1
TIM-3: T-cell immunoglobulin and mucin-domain containing-3
AMPK: AMP-activated protein kinase
NF-κB: Nuclear factor kappa-light-chain-enhancer of activated B cells
lncRNAs: Long non-coding RNAs
LINC01133: Long intergenic non-protein coding RNA 1133
DBP: Dibutyl phthalate
PDE4: Phosphodiesterase-4
MSCs: Mesenchymal stem cells
MRI: Magnetic resonance imaging.
